# Astrocytic timekeepers and their influence on brain waste clearance in Alzheimer's disease

**DOI:** 10.1016/j.nbscr.2026.100155

**Published:** 2026-10-13

**Authors:** Chun Lei Koenders, Roland Chiu, Tiago Medeiros-Furquim, Steven Bergink

**Affiliations:** University College Groningen, University of Groningen, Hoendiepskade 23, Groningen, 9718 BG, the Netherlands

## Abstract

Alzheimer's disease is associated with disruptions in circadian rhythmicity, yet the underlying mechanisms remain only partially understood. Developing insights suggest that astrocytes possess their own intrinsic circadian clocks, influencing multiple functions in brain homeostasis. Given the central role of astrocytes in brain waste clearance, this review investigates how the astrocytic clock mediates brain waste clearance and contributes to Alzheimer's disease pathology. By reviewing recent findings on extracellular clearance, cellular protein homeostasis, cellular detoxification/redox defence and neurotransmitter homeostasis, this review bridges research on astrocytic clock disruption with altered brain waste clearance dynamics. It further highlights the relevance of these alterations to Alzheimer's disease pathology. Interestingly, some clock-gene disruptions are associated with markers suggestive of increased autophagic activity and glymphatic-related gene expression, which may represent a compensatory response to circadian clock alteration. However, whether these changes translate into improved functional clearance *in vivo*, and whether they meaningfully affect Alzheimer's disease progression, remains uncertain. In contrast, similar clock-gene alterations generally impair blood-brain barrier integrity, glutamate clearance and antioxidant response, decreasing waste clearance capacity. These findings suggest a possible bidirectional relationship, in which some pathways may partially compensate for impairments in others. However, it remains unclear to what extent an imbalance in these pathways contributes to the development and/or progression of Alzheimer's disease. Altogether, the astrocytic circadian clock is positioned as an important regulator of brain waste clearance and warrants future mechanistic and therapeutic investigation.

## Highlights

• Connects the insights on circadian rhythms, astrocytes and Alzheimer's disease.
• Discusses protein clearance, neurotransmitter homeostasis, and redox defence.
• Provides insights on key knowledge gaps.

## 1 Introduction

Circadian rhythms regulate physiology, metabolism, and behaviour over time, allowing organisms to adapt to cyclic environmental changes such as the day and night cycle (Costa and Montagnese, 2021). Disturbances in these rhythms are often associated with pathology. In the most basic form, circadian rhythms are generated by a cell-autonomous transcription-translation negative feedback loop mechanism (TTFL) targeting a set of clock genes (Brancaccio et al., 2021). The master circadian clock is located in the suprachiasmatic nucleus (SCN), in the anterior hypothalamus (Brancaccio et al., 2021). In addition to this so-called master clock, several cell types have their own (cell-autonomous) circadian clock (Annamneedi et al., 2021; Hastings et al., 2019). Intriguingly, recent insights reveal an interplay between the circadian clock, astrocyte function, and Alzheimer's disease (AD). Disruptions in brain waste clearance mechanisms are associated with the accumulation of toxic proteins such as amyloid-β42 (Αβ42) and tau, key pathological features of AD (Knopman et al., 2021). It has become clear that astrocytes play a crucial role in brain waste clearance and maintaining the health of neurons (Oberheim et al., 2011). Several of the pathways/routes discussed below, including blood-brain barrier dysfunction, glutamate excitotoxicity, and oxidative stress, are not unique to AD but are shared across multiple neurodegenerative conditions. Because these pathways contribute to neurodegeneration, and astrocytes are suggested to regulate many of them, we focus here on AD as a disease context in which astrocyte dysfunction may influence disease progression. There is growing evidence suggesting that astrocytes possess autonomous circadian clocks themselves (Costa and Montagnese, 2021). This could suggest that astrocytes may link circadian timing to brain waste clearance capacity. Sleep/wake-state-dependent processes, including changes in arousal, posture, blood pressure and neuronal activity that occur regardless of circadian rhythm are known to impact brain waste clearance as well and have been linked to various neurodegenerative diseases (Gędek et al., 2023; Kylkilahti et al., 2021). Although these sleep/wake related effects are often intertwined with circadian rhythms, they are distinct and are discussed here mainly where they affect interpretation of clearance studies. Circadian disruption, astrocyte dysfunction and impaired brain waste clearance have each been linked to AD, but their intersection remains poorly integrated. We propose that astrocyte-intrinsic clocks coordinate multiple clearance and detoxification pathways and that disruption of this coordination may contribute to AD vulnerability and/or progression.

In this narrative review, we use the term *brain waste clearance* broadly to encompass the mechanisms by which the brain removes potentially harmful molecules and maintains cellular and extracellular homeostasis. This includes at least four distinct, though interrelated, categories: (1) extracellular clearance, such as glymphatic flow and blood-brain barrier (BBB) efflux that mediate the transport of Aβ42 and tau, (2) cellular protein homeostasis, including autophagy, (3) cellular detoxification and redox defence, including antioxidant responses that limit oxidative damage from reactive by-products, and (4) neurotransmitter homeostasis, particularly astrocytic glutamate uptake, which prevents excitotoxic accumulation. While glutamate regulation and antioxidant defence are not classically described as “waste clearance” in the same sense as Aβ/tau degradation or perivascular drainage, we include them here because astrocytic dysfunction in these domains contributes to the accumulation of neurotoxic species and thus to the same downstream burden the brain must clear. Each of the following sections addresses one of these four categories in the context of the astrocytic circadian clock and its relevance to AD.

## 2 Astrocytes

Glia (or neuroglia) reside among neural tissue (Hastings et al., 2023) and are a class of electrically non-excitable, non-neuronal cells. In the central nervous system, the main classes of glial cells are oligodendroglia, microglia, and astrocytes (Farhy-Tselnicker and Allen, 2018; Kimelberg and Nedergaard, 2010), with astrocytes accounting for the largest population of glial cells in the mammalian brain (Allen and Cagla, 2017).

Although they do not generate fast, propagating action potentials, astrocytes display a form of intracellular excitability mediated by Ca^2+^ signals, as well as Na^+^ and other second messengers, enabling them to sense and modulate neuronal activity. The foot processes of the astrocytes are spread across neural circuits where they contact up to thousands of synapses to regulate neuronal information processing at the local synapse but also circuit-level through direct contact or the secretion of gliotransmitters (Farhy-Tselnicker and Allen, 2018; Hastings et al., 2023). Moreover, astrocytes form an interconnected network through gap junctions, allowing them to coordinate their responses to stimuli (Vasile et al., 2017). Although astrocytes have long been considered mere support cells to neurons, research over the past decades has shown otherwise (Jäkel and Dimou, 2017; Oberheim et al., 2011). For example, in both *in vivo* and cultured neurons, astrocyte ablation resulted in neuronal degeneration and eventually cell death, highlighting their importance (Jäkel and Dimou, 2017; Oberheim et al., 2011). Essential central nervous system functions such as metabolism, structure, homeostasis, and neuroprotective tasks are all under astrocytic regulation (Oberheim et al., 2011).

### 2.1 The synapse and neurotransmitters

Through the tripartite synapse, a synapse composed of the pre- and postsynaptic neurons and an astrocyte, the astrocytes mediate neuronal signalling (Hastings et al., 2023). Tight regulation, including clearance of the excitatory neurotransmitter glutamate and inhibitory neurotransmitter gamma aminobutyric acid (GABA), is required to maintain synaptic homeostasis and prevent excitotoxicity (Carello-Collar et al., 2023; Chi-Castañeda and Ortega, 2018; Mahmoud et al., 2019). This tight regulation is facilitated by glutamate and GABA transporters (Mahmoud et al., 2019). Notably, 80-90% of the overall glutamate uptake in the brain is performed by astrocytic glutamate transporters, GLAST and GLT-1, also known as EAAT-1 and EAAT-2, respectively (Chi-Castañeda and Ortega, 2018). They take up glutamate into the astrocytes, where it is converted into glutamine via glutamine synthetase (He et al., 2018). This conversion, called the glutamate-glutamine cycle, is required to prevent excitotoxicity during transport back to the neurons (He et al., 2018; Rudy et al., 2015). In the neurons, it will be converted back to glutamate (He et al., 2018). Another mechanism of astrocytes modulating neuronal signalling is through K^+^-buffering (Bellot-Saez et al., 2017). Additionally, astrocytes act as energy reservoirs, storing glycogen and supplying neurons with metabolic substrates through the astrocyte-neuron lactate shuttle, which produces lactate for alternative energy sources (Bezzi and Volterra, 2011; Mason, 2017). Lastly, astrocytic endfeet are part of the blood-brain barrier, maintaining its integrity and mediating solute transport (Gawdi et al., 2023). Although these functions appear to be distinct, they all contribute to maintaining brain homeostasis and limiting the accumulation of potentially harmful molecules.

### 2.2 The circadian clock and astrocytes

As the brain is expected to perform different tasks during daytime compared to nighttime (sensory and motor engagement versus sleep, memory consolidation, and repair), tight regulation of the clock is essential (Hastings et al., 2023). Interestingly, astrocytes have rhythmic oscillations in their clock genes and thus a clock output (Costa and Montagnese, 2021; Jackson et al., 2019). On the molecular level, ablation of the clock gene, *Bmal1,* in SCN astrocytes was shown to lengthen the circadian period of both locomotor behaviour as well as clock gene, *Per2*, expression in the SCN (Tso et al., 2017). Additionally, *Cry1/2*-null mice lack behaviour rhythmicity; however, astrocyte-specific expression of *Cry1* rescues the circadian behaviour even in the absence of *Cry* expression in neurons (Brancaccio et al., 2019). This is also reflected at the functional level, as *Cry1/2-*deficient mice demonstrated no detectable circadian oscillations of extracellular glutamate levels, while WT mice show clear oscillations (Brancaccio et al., 2019). Importantly, rescue with *Cry*-expressing astrocytes was enough to restore these oscillations (Brancaccio et al., 2019). Moreover, neurons in the SCN are predominantly GABAergic (Brancaccio et al., 2019, 2021), and the extracellular glutamate oscillations are antiphasic to neuronal activity while in-phase with astrocytic activity (Brancaccio et al., 2019), suggesting that the rhythmic oscillations in extracellular glutamate levels originated from astrocytes (Brancaccio et al., 2019).

Notably, astrocytes are involved in the tripartite synapse between the retinohypothalamic tract and the “first” SCN neurons (Costa and Montagnese, 2021). Due to this position and the functionality of astrocytes, it has been hypothesized that they play a part in mediating circadian timekeeping of the master clock's output (Barca-Mayo et al., 2017; Brancaccio et al., 2017). The studies of Brancaccio et al. (2017) and Barca-Mayo et al. (2017) supported this notion, highlighting the importance of correct glutamate and GABA signalling, respectively. In pan-astrocytic *Bmal1*-deleted mice, cortical GABA transporter expression is downregulated while extracellular levels are elevated, preventing the synchronisation of neurons via astrocytes (Barca-Mayo et al., 2017; Hastings et al., 2023). The absence of synchronisation of these neurons is reflected in an altered circadian and cognitive phenotype of the mice (Barca-Mayo et al., 2017). In general, neurons rely on glutamatergic signalling of astrocytes, and more specifically, astrocytes are responsible for the metabolic activity during nighttime, while neurons are more active during the daytime (Brancaccio et al., 2017), supporting the notion that SCN timekeeping is a product of interdependent and mutually supportive astrocyte-neuronal interactions. The intracellular calcium levels of astrocytes show antiphasic oscillations to neurons, while being in phase with extracellular glutamate levels (Brancaccio et al., 2017). The extracellular glutamate in the SCN acts on glutamate receptors of multiple cell types, including the GABAergic neurons (Brancaccio et al., 2017). As an excitatory neurotransmitter, high extracellular levels will lead to activation and depolarisation of GABAergic neurons (Brancaccio et al., 2017). Subsequently, this causes an overall inhibitory effect on the SCN network's metabolic activity during the night (Brancaccio et al., 2017). These findings point to a firm role of astrocytes on the circadian clock and reveal how circadian rhythms control astrocyte function. This relationship matches transcriptional needs with the daily cycles of physiology in the brain.

## 3 Brain waste clearance and Alzheimer's disease

The brain is a high energy-consuming organ and releases a substantial amount of potentially toxic metabolic by-products, such as CO_2_, lactate, and potentially hazardous proteins (Kaur et al., 2021). Therefore, rapid waste clearance mechanisms are crucial to avoid their accumulation (Kaur et al., 2021), especially of misfolded or aggregated proteins, which need to be removed to avoid toxicity and accumulation (Ihara et al., 2012; Kampinga and Bergink, 2016).

In addition to extracellular protein clearance, astrocytic regulation of neurotransmitter homeostasis, particularly glutamate uptake, represents a distinct form of waste management relevant to AD. Neurodegenerative diseases, such as AD, are accompanied by an excess of extracellular glutamate (Wang and Reddy, 2016). This so-called excitotoxicity causes neuronal hyperexcitation, which eventually leads to neuronal death (Mahmoud et al., 2019), resulting in the cognitive impairments associated with AD (Keimasi et al., 2023). Meta-analyses have shown a significantly lower level of GABA in the cerebrospinal fluid of the AD brain compared to healthy individuals, indicating a reduced ability to inhibit excitatory neuronal activity (Carello-Collar et al., 2023). Moreover, the glutamatergic systems show significantly decreased uptake (Soares et al., 2024). Together, these findings suggest increased glutamate levels in the extracellular space of AD patients (Carello-Collar et al., 2023; Soares et al., 2024).

As glutamate transporters regulate these levels (Mahmoud et al., 2019), inhibiting the synthesis of GLAST and GLT-1 transporters, in rats and *in vitro* models, results in elevated extracellular glutamate levels to the point of neurotoxicity with its neurodegenerative characteristics (Rothstein et al., 1996). Restoring GLT-1 has beneficial effects on AD pathology, including improved cognitive function, reduced Aβ42-plaques, and restored synaptic integrity (Takahashi et al., 2015). Conversely, Aβ42 has been shown to downregulate GLAST and GLT-1 expression, which could decrease astrocytic uptake of glutamate (S. Huang et al., 2018). Notably, most of the evidence discussed here focuses on Aβ42 specifically; however, the Aβ42/Aβ40 ratio, rather than Aβ42 alone, is increasingly used as a more reliable biomarker of AD pathology in both CSF and plasma.

A well-studied risk factor in AD is the polymorphic protein apolipoprotein E (ApoE, ApoE4 and ApoE2, risk and protection, respectively) (Jackson et al., 2024; Kim et al., 2009). ApoE is produced predominantly by astrocytes and facilitates the transfer of cholesterol and phospholipids between cells. (Liao et al., 2016; Zhou et al., 2023). High ApoE4 expression in astrocytes can disrupt cerebral cholesterol homeostasis, contributing to neurotoxic downstream effects associated with an AD phenotype (Zhou et al., 2023).

### 3.1 Circadian rhythm in brain waste clearance

Recent findings suggest that Aβ42 clearance is impaired in AD and potentially contributes to the accumulation of plaques in the extracellular space (Knopman et al., 2021; Wildsmith et al., 2013). Through perivascular efflux, Aβ42 is transported across the blood-brain barrier and/or Aβ42 is degraded by glial cells, including astrocytes (Knopman et al., 2021). Interestingly, the clearance of Aβ42 seems to be under clock regulation as it undergoes circadian oscillations (Clark et al., 2022; Schurhoff and Toborek, 2023). Disruption of circadian regulation could therefore contribute to the accumulation of the Aβ42. Notably, disturbances in circadian and sleep/wake patterns, including fragmented sleep, increased wakefulness, and reduced daytime activity, occur in early and even preclinical stages of AD (Knopman et al., 2021; Yang et al., 2025).

Although extracellular glutamate levels show circadian oscillations, there is an absence of circadian fluctuations in glutamate uptake (Beaulé et al., 2009; Leone et al., 2015); rather, it shows diurnal variation (Leone et al., 2015). This suggests that glutamate uptake may be influenced by exogenous light cues (Leone et al., 2015). However, the circadian regulation of glutamate uptake could not be ruled out (Chi-Castañeda and Ortega, 2018). Despite this, astrocytes may still exert indirect circadian control through their role in the tripartite synapse, specifically at the retinohypothalamic tract synapse, where light information enters (Costa and Montagnese, 2021). Astrocytes have been shown to regulate extracellular glutamate levels in a circadian manner (Brancaccio et al., 2017, 2021), suggesting an interplay between endogenous clocks and external cues.

Notably, the mRNA and protein levels of GLAST show a diurnal rhythm in natural conditions (Spanagel et al., 2004). Although entrained by light, a contribution of clock-gene-regulation is suggested by reduced GLAST expression and astrocytic glutamate uptake in mice with mutated clock genes (Beaulé et al., 2009; Spanagel et al., 2004).

Apart from glutamate transporters, other mechanisms, including the gap junction connexin43 (CX43) and the enzyme glutamine synthetase, may contribute to the circadian fluctuations in extracellular glutamate levels, as CX43 expression is under astrocyte-specific circadian control (Ali et al., 2019; Negoro et al., 2012). Inhibition of CX43, which could be achieved through astrocytic clock alterations, leads to disrupted extracellular glutamate oscillations (Brancaccio et al., 2019). However, whether clock alterations affect CX43 sufficiently to disrupt these glutamate fluctuations remains unclear.

The abundance and activity of glutamine synthetase, a component regulating glutamate levels, also exhibit circadian fluctuations (Leone et al., 2015). This is consistent with endogenous regulation, with higher levels during the (subjective) day (Leone et al., 2015). Deficient availability of glutamine synthetase in astrocytes could be a potential mechanism underlying extracellular glutamate accumulation (Eid et al., 2004; Zou et al., 2010).

Because astrocytes regulate extracellular glutamate levels (Brancaccio et al., 2021), alterations in the astrocytic clock may impair glutamate regulation through impairing glutamate uptake (Brancaccio et al., 2017), glutamine synthetase (Leone et al., 2015), and CX43 (Brancaccio et al., 2019), contributing to excitotoxicity. Given glutamate's centrality to cognition and neurotoxicity, it has a major impact on AD pathology, potentially exacerbating its phenotype (Mahmoud et al., 2019; R. Wang and Reddy, 2016). These findings support further investigation of whether targeting astrocytic clock mechanisms could restore glutamate homeostasis.

Brain waste products can be cleared via the blood-brain barrier or the glymphatic system or processed by brain cells including glial and neurons. Astrocytes contribute to both extracellular transport and cell autonomous clearance mechanisms.

## 4 The blood-brain barrier

The removal of waste products in the brain depends on several mechanisms, including transport across the blood-brain barrier, which lines the brain's blood vessels (Fig. 1; see Ding et al., 2023 for more information) (Ding et al., 2023; Kadry et al., 2020; Zhao et al., 2022). Integrity loss of the blood-brain barrier can lead to impairment of waste clearance, which has implications in several neurodegenerative diseases, including AD (Kadry et al., 2020).

**Fig. 1:**
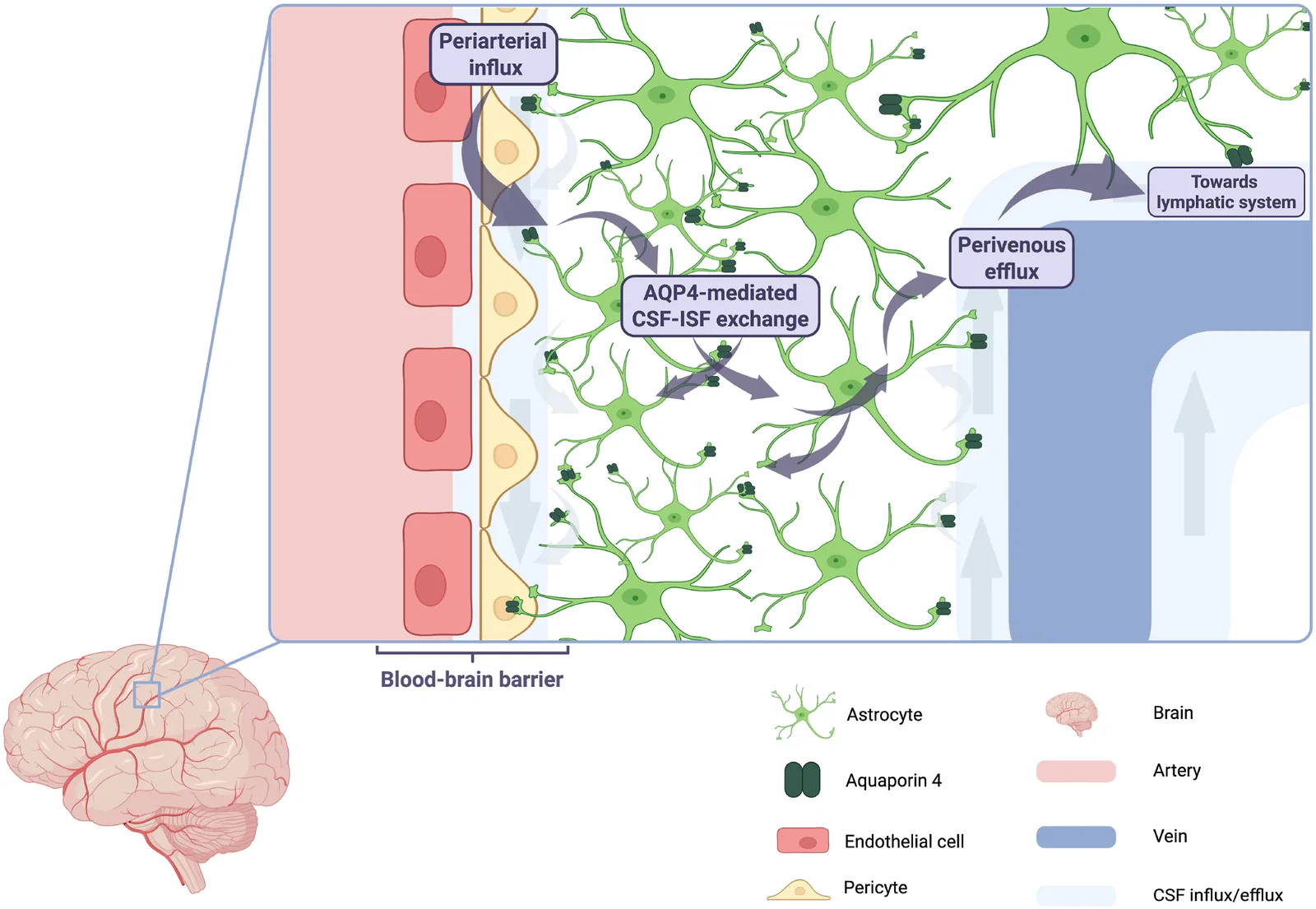
**Representation of the blood-brain barrier and the glymphatic system.** The blood-brain barrier (BBB) is primarily composed of endothelial cells, pericytes and astrocytic endfeet, which together form a highly selective structure that restricts the entry of neurotoxic substances into the brain parenchyma. Astrocytic endfeet additionally contribute to the glymphatic system by facilitating clearance of metabolic waste from the interstitial space. Through the expression of aquaporin-4 (AQP4) channels on the astrocytic endfeet, cerebrospinal fluid (CSF) and interstitial fluid (ISF) are exchanged within the interstitium, promoting convective waste transport. Metabolic waste products are subsequently drained into the perivenous space, where they travel toward the lymph nodes for clearance.

The blood-brain barrier is composed of specialised endothelial cells, mural cells (i.e., pericytes and smooth muscle cells) and the basement membrane (McConnell and Mishra, 2022). It also functions as part of the neurovascular unit (NVU), a structure consisting of neurons, astrocytes and microglia (McConnell and Mishra, 2022; Zhao et al., 2022). Together, through coordinated intracellular signalling, these components form a highly selective, semipermeable barrier that restricts the influx of unwanted materials and neurotoxins from the bloodstream into the CNS (Gawdi et al., 2023). The crossing of molecules over the blood-brain barrier is regulated by tight junctions, which are the major mediators of sustaining the semi-permeability, by physically restricting molecules and cells from passively crossing the border (McConnell and Mishra, 2022; Wu et al., 2023).

Notably, astrocytes contribute to the neurovascular unit through endfeet that closely contact the blood-brain barrier (McConnell and Mishra, 2022; Schurhoff and Toborek, 2023). Due to their location between the bloodstream and the brain, they are the first place of contact with solutes (McConnell and Mishra, 2022; Schurhoff and Toborek, 2023). They regulate ionic concentrations and protein transporters through specialised channels like aquaporin-4 (AQP4) (Cabezas et al., 2014), coordinating their responses through connexin (CX) 30 and CX43 gap junctions (Cabezas et al., 2014; Yin et al., 2018).

Due to the blood-brain barrier's importance in the tight regulation of homeostasis, its dysregulation can have detrimental effects, as seen in AD (Kadry et al., 2020). Blood-brain barrier integrity loss is a hallmark associated with cognitive decline (Nehra et al., 2022). AQP4 deletion in Alzheimer's murine models has been shown to increase Aβ42 burden, decrease its clearance, as well as exacerbate cognitive deficits, while postmortem human samples show altered AQP4 localisation (Simon et al., 2022; Xu et al., 2015). Mislocalisation of AQP4 was a common phenotype of the AD samples (Xu et al., 2015), affecting the activity and effectiveness of AQP4 (Silva et al., 2021).

The major AD risk factor ApoE4 is implicated in blood-brain barrier impairments (Zhou et al., 2023). The neurotoxic state caused by its high expression leads to oxidative stress (Zhou et al., 2023). It promotes blood-brain barrier integrity impairment through the contraction of endothelial cells, resulting in reduced tightness of tight junctions (Kim et al., 2024; Zhou et al., 2023), as well as reduced astrocyte endfoot coverage of blood vessels (Jackson et al., 2021; Nishitsuji et al., 2011). The removal of this astrocyte-produced ApoE4 ameliorates these phenotypes (R. J. Jackson et al., 2021; Nishitsuji et al., 2011).

### 4.1 Circadian rhythm and the blood brain barrier

Interestingly, the above-mentioned factors of the blood-brain barrier are shown to follow a circadian rhythm. Tight junction proteins and apolipoproteins have been reported to be subjected to circadian oscillations (Lee et al., 2023; McKee et al., 2022; Oh-oka et al., 2014). The study of McKee et al. (2022) showed that astrocyte-specific knock-out of *Bmal1* results in significant upregulation of *ApoE*. This suggests a potential link between astrocytic BMAL1-dependent regulation of ApoE4 and AD pathology, although it remains unclear whether this reflects circadian disruption itself.

Additionally, AQP4 is found to support the circadian rhythm of water transport in the brain, thereby facilitating oscillations of ion concentrations (Hablitz et al., 2020). Moreover, localisation of AQP4 channels shows changes in a circadian fashion (Hablitz et al., 2020). Astrocyte-specific *Bmal1* knock-out was shown to elevate expression of *Aqp4* mRNA (Lananna et al., 2018). However, the absence of BMAL1 in neurons and glia causes cortical AQP4 levels to decrease (Nakazato et al., 2017). These findings present conflicting results, which could implicate regulation at multiple levels. It could very well be that increased *Aqp4* expression does not translate into an increase in protein levels, seen as cortical AQP4 levels decrease (Nakazato et al., 2017). Additionally, it could not result in correct localisation to the astrocytic endfeet, which are ultimately responsible for increasing glymphatic flow (Silva et al., 2021). The research showing decreased AQP4 levels is performed in astrocytes and neuron-deleted *Bmal1* mice (Nakazato et al., 2017), while the research demonstrating an increase in *Aqp4* levels is done in astrocyte-specific *Bmal1* mice (Lananna et al., 2018). This could suggest a distinct difference in the mechanisms of astrocytic and neuronal clocks. It may be that astrocytic deletion is protective (increasing *Aqp4*), and that neuronal deletion causes the decrease in AQP4. However, more research is needed to explore this.

Lastly, the ABC transporter permeability-glycoprotein (P-gp) was found to be under circadian control (Cuddapah et al., 2019; Kervezee et al., 2014). P-gp mediates the efflux of endogenous and exogenous molecules across the blood-brain barrier, enhancing the barrier's selectivity (Kervezee et al., 2014). Loss of the circadian clock resulted in constant expression of P-gp (Kervezee et al., 2014). This could be seen as a protective mechanism as higher expression is associated with increased Aβ clearance (Wang et al., 2016). However, more studies are needed to clarify whether alterations in P-gp contribute significantly to AD pathology.

Serving as the primary interface between the systemic circulation and the brain, the maintenance of the blood-brain barrier integrity is crucial to prevent AD pathogenesis (Kadry et al., 2020). Disturbances of the astrocytic clock have been demonstrated to impair blood-brain barrier function through tight junctions (Oh-oka et al., 2014) and alter CX43 signalling (Ali et al., 2019). These changes could reduce brain waste clearance and increase the influx of neurotoxins (Nehra et al., 2022). Given that the early and progressive breakdown of the blood-brain barrier observed in AD (Nehra et al., 2022) aligns with the early presenting circadian disruptions in the early phases of AD (Knopman et al., 2021), circadian regulation of blood-brain barrier function may have an influence on the disease progression. However, interpretation of these findings requires caution, as many insights are gained specifically from *Bmal1* knockout mice models. It remains unclear whether these mechanisms are regulated by the circadian clock or by BMAL1 itself, whose effects extend beyond timekeeping. Although *Bmal1* is a core clock gene, its regulatory function can reflect normal transcriptional control rather than circadian rhythmicity (McKee et al., 2023; Zheng et al., 2023). Nonetheless, the indirect effect of BMAL1 should not be overlooked. These possibilities raise questions about straightforward interpretations and show the importance of isolating circadian-specific regulation from gene-specific regulation when assessing whether targeting the astrocytic clock, and specifically P-gp transport, offers a therapeutic target for preserving blood-brain barrier function, mitigating AD pathology.

## 5 Glymphatic system

Considering the large distance between the interstitial solutes and the blood-brain barrier and the selectivity of the blood-brain barrier, other clearance pathways are favoured including astroglial-mediated fluid flow (Silva et al., 2021). The aquaporin-4 (AQP4) channels present on the astroglial endfeet facilitate the exchange of cerebrospinal fluid and interstitial fluid into the interstitium (Iliff et al., 2012). This system, also known as the glymphatic system, can clear metabolic waste from the interstitial space (Fig. 1) (see Gao et al., 2023 for more information) (Gao et al., 2023; Iliff et al., 2012; Verheggen et al., 2018; E. A. Yamamoto et al., 2024). This clearance pathway involves several distinct but sequential steps: periarterial cerebrospinal fluid (CSF) influx, AQP4-facilitated CSF–interstitial fluid (ISF) exchange across astrocytic endfeet, bulk interstitial solute flow, and perivenous efflux into the meningeal lymphatic vessels, which ultimately drain into cervical lymph nodes. Because AQP4 mediates only the CSF–ISF exchange step, changes in AQP4 expression or localisation do not necessarily reflect changes in the downstream steps of perivascular drainage or meningeal lymphatic clearance.

The impairment of the glymphatic system, and specifically AQP4, has been implicated in AD (Silva et al., 2021). Insufficient flow through the AQP4 channels of the glymphatic system has been associated with Aβ42 accumulation, possibly increasing the risk of AD (Nedergaard and Goldman, 2020; Silva et al., 2021). Conversely, enhanced Aβ removal through the glymphatic system has been associated with reduced AD-related pathology (Li et al., 2024).

### 5.1 Circadian rhythm and the glymphatic system

Notably, the glymphatic system has been shown to undergo fluctuations throughout the day, suggesting circadian control (Hablitz et al., 2020). Although much of the literature suggests that the sleep-wake cycle regulates glymphatic clearance (Cuddapah et al., 2019; Xie et al., 2013), emerging evidence also supports a contribution of circadian regulation to glymphatic function (Cai et al., 2019; Hablitz et al., 2020; Yan et al., 2021). As AQP4 is the key mediator of glymphatic system functionality (Silva et al., 2021), its contribution to the glymphatic system's circadian regulation is perhaps unsurprising. Much of the literature on glymphatic clearance (e.g., Xie et al., 2013; Kang et al., 2009) measures Aβ or CSF–ISF exchange across the sleep-wake cycle, making it difficult to isolate a purely circadian contribution. Experimental paradigms that impose forced wakefulness or sleep at varying circadian phases (Cai et al., 2019) offer one route to dissociating these factors, but such designs remain rare.

The glymphatic system plays an important role in clearing brain waste into the perivascular space, essential for neural homeostasis (Silva et al., 2021). Astrocyte-specific Bmal1 deletion has been associated with increased Aqp4 expression (Lananna et al., 2018), although whether this enhances functional glymphatic clearance remains unclear. However, elevated mRNA levels do not directly translate into increased protein levels (Nakazato et al., 2017) or correct localisation of AQP4 channels, which is ultimately responsible for glymphatic function. Additionally, the efficiency of the glymphatic system is closely tied to the integrity of the BBB (Silva et al., 2021), which is often compromised in AD (Nehra et al., 2022). Therefore, while the glymphatic system contributes to brain waste clearance, its modulation by the astrocytic clock and its importance to AD pathology remains uncertain.

## 6 Autophagy

Cellular clearance of unnecessary proteins mostly happens through the ubiquitin-proteasome and/or the autophagy-lysosome systems (Metcalf et al., 2010). Autophagy delivers unwanted and damaged cellular content to the lysosome for degradation (Yamamoto et al., 2023). It therefore contributes substantially to cellular protein homeostasis and intracellular clearance. There are three dominant types of autophagy, including macroautophagy, chaperone-mediated autophagy (CMA) and microautophagy (Parzych and Klionsky, 2014). Microautophagy is less well characterised in AD, and therefore the focus will be on macroautophagy and CMA (Fig. 2; see Litwiniuk et al., 2023 for more information; Litwiniuk et al., 2023; Ntsapi and Loos, 2021).

**Fig. 2:**
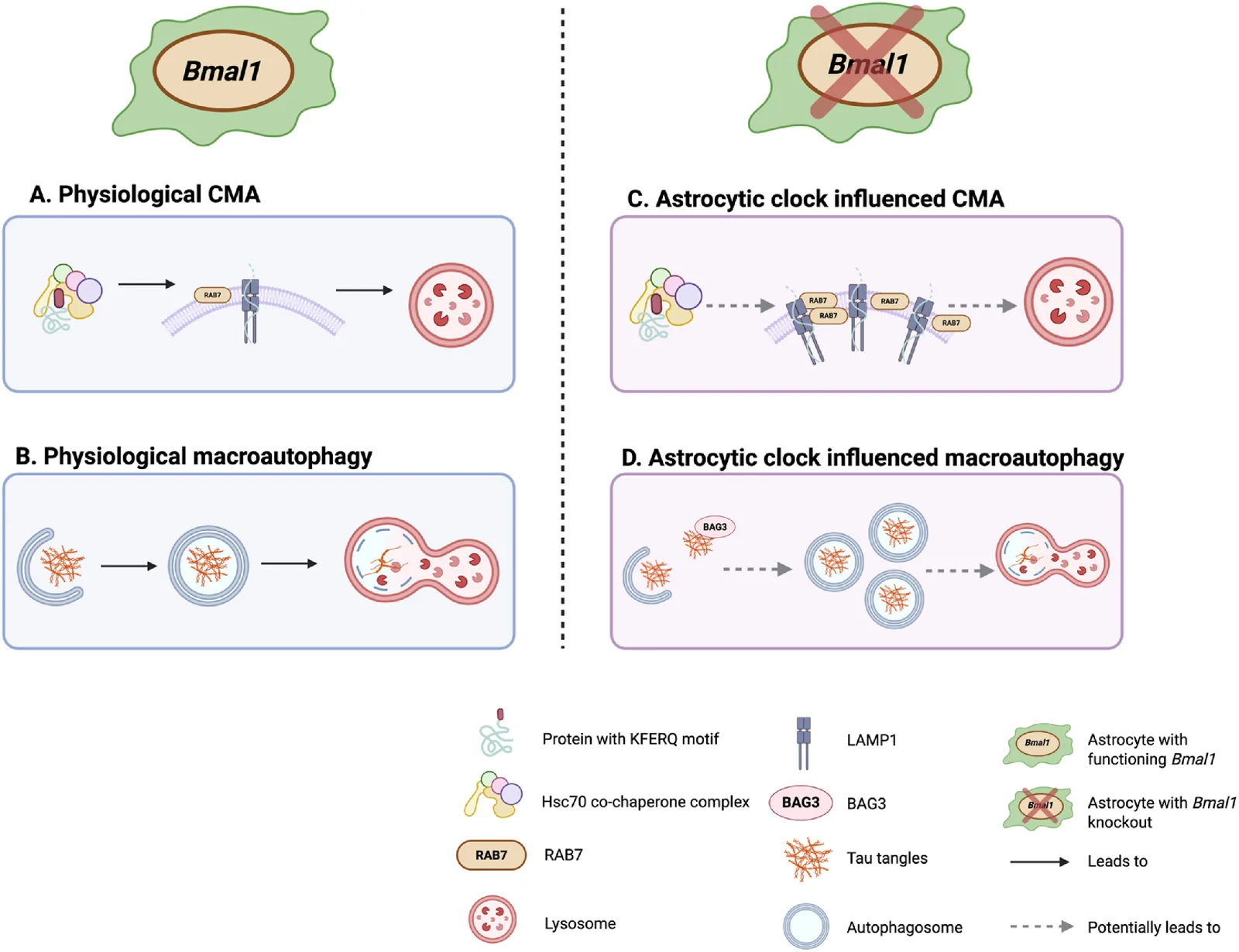
**Astrocytic *Bmal1* knockout is associated with chaperone-mediated autophagy (CMA) and macroautophagy.** In normal astrocytes (on the left), CMA is initiated by recognition of cytosolic proteins containing KFERQ-like motifs by HSC70, followed by lysosomal translocation through LAMP2A receptor and subsequent degradation within the lysosome. Macroautophagy proceeds through initiation and autophagosome formation, followed by fusion with lysosomes to generate autolysosomes for cargo degradation. In *Bmal1* knockout astrocytes (on the right), CMA activity is associated with increased substrate uptake, elevated HSC70-mediated delivery, increased LAMP1 and RAB7 expression and increased lysosomal degradation activity. Macroautophagy is similarly associated with BAG3-dependent activation, potentially contributing to increased autophagosome formation, autolysosome fusion and degradation of aggregated proteins.

The first stage of the macroautophagy pathway is mediated by AMP-activated protein kinase (AMPK) and mammalian target of rapamycin complex 1 (mTORC1) kinase (Ortega et al., 2024; Sung and Jimenez-Sanchez, 2020). Normally, mTORC1 suppresses initiation under nutrient-rich conditions, but upon nutrient and energy depletion, mTORC1 becomes inactivated and dissociates from the complex while AMPK triggers autophagy (Barmaki et al., 2023). Subsequently, the dedicated autophagy pathway targets particular substrates and proteins to the developing autophagosomes (Barmaki et al., 2023). A critical step is the activation of LC3-I. Once LC3-I has been targeted to the autophagosomal membrane, it gets activated to become conjugated to phosphatidylethanolamine and is called LC3-II (Sung and Jimenez-Sanchez, 2020). The autophagosome continues to elongate, matures and fuses with the lysosome, where its content will be degraded (Barmaki et al., 2023; Sung and Jimenez-Sanchez, 2020).

Chaperone-mediated autophagy, on the other hand, selectively degrades proteins containing KFERQ-like motifs (Yao and Shen, 2023). This motif is recognised by the heat shock cognate protein 70 (HSC70), which delivers the substrate protein to the lysosomal membrane (Yao and Shen, 2023).

### 6.1 Implications in Alzheimer's disease

Autophagy has been implicated in AD. For example, impaired autophagy leads to insufficient clearance of Aβ42 and tau aggregates, which both exacerbate accumulation and increase neurotoxicity (Barmaki et al., 2023). Moreover, findings that enhanced autophagy can reduce AD-related pathology (McKee et al., 2019; Xiao et al., 2014) support further investigation of autophagy as a potential drug therapeutic target.

The molecular mechanism behind a potential role of autophagy in AD is still unclear. CMA deficiency is implicated in AD pathogenesis (Caballero et al., 2017, 2021). Wild-type tau is predominantly targeted by the CMA pathway (Caballero et al., 2017). Acetylated tau, a pathological tau species observed in AD, is preferentially targeted by the macroautophagy pathway (Caballero et al., 2017). Caballero et al. (2021) suggests that this may result from acetylated-tau-induced inhibition of CMA, although cause and effect remain difficult to establish. Notably, amyloid beta precursor protein (APP) has a KFERQ motif on its C-terminus but was shown not to be targeted by CMA as deletion of this motif does not change full-length APP levels (Park et al., 2016). Instead, APP is mainly degraded by macroautophagy (Park et al., 2016). However, in the presence of high levels of APP and/or Aβ, both macro- and chaperone-mediated autophagy are upregulated (Ntsapi and Loos, 2021).

Astrocytes’ autophagy dysfunction is especially implicated in AD progression (McKee et al., 2023). Reactive astrocytes surround amyloid plaques and internalise Aβ to subsequently perform autophagy-based degradation (Gomez-Arboledas et al., 2017; Ries and Sastre, 2016; Wyss-Coray et al., 2003). Furthermore, ApoE4, a major genetic risk factor for AD (R. J. Jackson et al., 2024), has been associated with reduced autophagy-dependent clearance of extracellular Aβ-42 compared with other ApoE isoforms (Simonovitch et al., 2016).

### 6.2 Circadian rhythm and autophagy

Interestingly, there are several studies suggesting circadian clock control of autophagy (Kang et al., 2009; Kress et al., 2018). Soluble Aβ peptides show oscillations in phase with circadian rhythmic activity (Kang et al., 2009). The research of Kress et al. (2018) supported this notion by demonstrating the disruption of interstitial fluid Aβ42 fluctuations upon global deletion (including the SCN) of *Bmal1* in the hippocampus. It could be that this is due to autophagy being under circadian control, as autophagy is important for the removal of Aβ42 peptides (Lee et al., 2015). Moreover, impaired autophagy mechanisms exacerbate Aβ42 levels (Barmaki et al., 2023). However, further research should take the next step by identifying if autophagy is indeed contributing to the Aβ42 fluctuations.

As mentioned previously, ApoE expression is regulated by the circadian clock (Kress et al., 2018). An increase in ApoE4 expression in AD patients carrying this isoform, due to circadian clock changes, could lead to reduced autophagy-dependent Aβ-42 clearance.

Notably, *in vitro* astrocytes with a *Bmal1* deficiency exhibit, alongside increased endocytosis and lysosome-dependent protein cleavage, accumulation of LAMP1 and RAB7, proteins defining the late lysosome (and endosome) important for CMA (McKee et al., 2023). BMAL1 deficiency is therefore associated with increased markers of lysosomal and autophagic activity, which may represent an adaptive response. However, the control was shown in a non-rhythmic manner (McKee et al., 2023). This could be due to BMAL1 supporting basal activity of CMA-related pathways rather than driving rhythmic expression. Therefore, it remains unclear whether a loss of BMAL1 leads to increased clearance and thus a diminished AD phenotype.

Macroautophagy is also suggested to be under astrocytic circadian clock regulation through Bcl2-associated athanogene 3 (BAG3), an essential chaperone for macroautophagy (Sheehan et al., 2023). The research of Sheehan et al. (2023) demonstrates that astrocyte-specific deletion of *Bmal1* activates *Bag3*. Consequently, this results in decreased tau pathology, shown to be due to increased clearance of the protein aggregates through macroautophagy in a *Bag3*-dependent manner (Sheehan et al., 2023). AD patients were shown to have increased *Bag3* expression in astrocytes with disease-associated phenotype, which could suggest an attempt to enhance clearance capacity in response to increased protein aggregation seen in AD (Sheehan et al., 2023). This enhancement was also reflected as astrocytic-specific *Bmal1* knockout increases autophagosomes (McKee et al., 2023). This finding, again, suggests an adaptive response as seen in CMA. However, only tau pathology was mitigated and tau pathology is thought to be downstream of Aβ42 accumulation (Aksman et al., 2023; Bloom, 2014; Gulisano et al., 2018). This suggests that the induced autophagy might have limited therapeutic effects as it fails to address Aβ42 plaques. Since BMAL1 regulates BAG3 and lysosomal/autophagic gene expression through mechanisms that may not be strictly rhythmic (McKee et al., 2023), the observed increase in autophagic clearance following *Bmal1* deletion may reflect a clock-gene-dependent rather than a circadian-rhythm-dependent effect.

Although these results suggest a compensatory mechanism to counteract dysfunctions of other mechanisms, previous research has shown that mTOR activity increases in *Bmal1* knockout mice, which suggests a decrease in autophagic activity rather than an increase (Khapre et al., 2014). A possible explanation for this discrepancy is that although Aβ42 uptake changes upon astrocyte-specific *Bmal1* knockout, overall Aβ42 plaque burden remains unchanged (McKee et al., 2022), suggesting that increased autophagic markers do not necessarily translate into enhanced Aβ42 clearance.

The maintenance of protein homeostasis performed by autophagy is crucial to prevent AD pathogenesis (McKee et al., 2023). Increase in both CMA and macroautophagy upon clock disruption suggests that autophagy could work as a protective compensatory mechanism to increase brain waste clearance when other mechanisms fail (McKee et al., 2023; Sheehan et al., 2023). The astrocytic clock appears to mediate this activity, with *Bmal1* emerging as a potential therapeutic target to increase brain waste clearance capacity (McKee et al., 2023; Sheehan et al., 2023). However, as AD is characterised by impaired autophagic machinery, the protective role of enhanced autophagy upon astrocytic clock disruption may only moderately impact disease outcomes, unable to outweigh the clock-induced neurotoxic effects. Moreover, there is a bidirectional relationship complicating interpretation. The circadian clock is known to regulate autophagic activity, but autophagic activity also influences the clock mechanism by degrading key circadian clock proteins and regulators (Di et al., 2023). This highlights the difficulty in distinguishing between the cause and effect of the astrocytic circadian clock. While autophagy's role is strongly implicated in AD pathology, whether its modulation via the astrocytic clock has a similar significant impact remains unclear.

## 7 Oxidative stress and its antioxidant defence

Oxidative stress is characterised by cell damage due to an imbalance of reactive oxygen species (ROS) and the defence system that eliminates these products (Huang et al., 2016). ROS is the umbrella term for all the highly reactive molecules derived from oxygen and includes free radicals as well as non-radical species, such as hydrogen peroxide (Averill-Bates, 2023). ROS are by-products of normal cellular metabolism; their physiological level is required for cell differentiation, apoptosis and modulation of signalling pathways (Jomova et al., 2023). This is called “good stress” or also known as oxidative eustress (Jomova et al., 2023). However, high levels of ROS/RNS can be toxic and lead to oxidative stress, which is harmful for cells (Fig. 3) (see Hammad et al., 2023 for more information) (Hammad et al., 2023; Jomova et al., 2023). To maintain homeostasis, antioxidants are present to neutralise and attenuate the harmful effects of oxidative stress (Jomova et al., 2023).

**Fig. 3:**
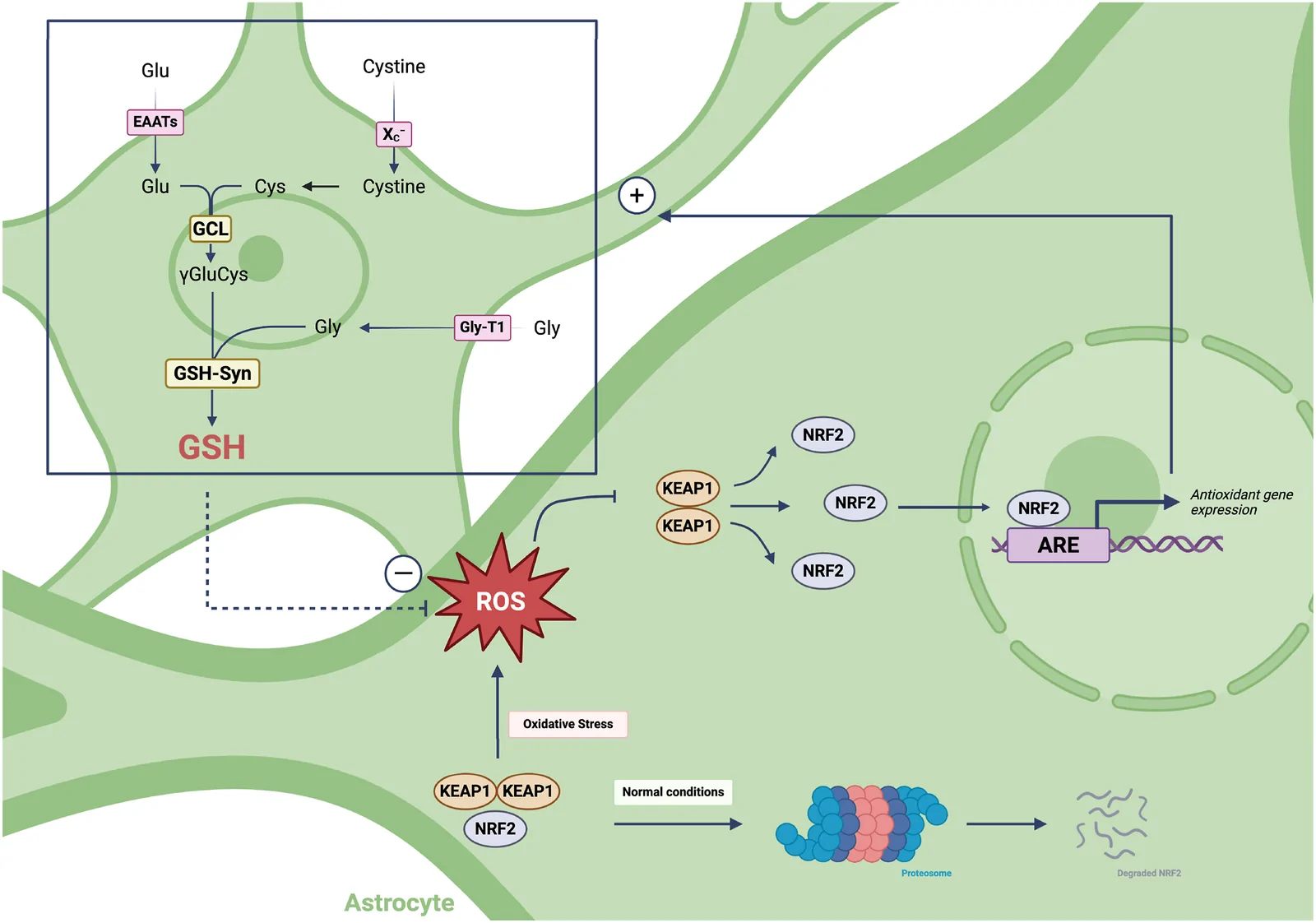
**A schematic overview of the interplay between the NRF2/ARE axis and GSH synthesis.** Under physiological conditions, Kelch-like ECH-associated protein 1 (KEAP1) binds and promotes degradation of nuclear factor erythroid 2-related factor 2 (NRF2), thereby limiting antioxidant gene transcription. During oxidative stress, reactive oxygen species (ROS) modify Keap1, preventing NRF2 degradation and enabling its translocation into the nucleus. Here, NRF2 binds to antioxidant response elements (ARE), activating transcription of antioxidant and detoxification genes. Among these are glutamate-cysteine ligase (GCL) and glutathione synthetase (GSH-syn), key enzymes involved in glutathione (GSH) synthesis. Increased GSH production contributes to neutralisation of ROS and restoration of redox homeostasis, thereby forming a feedback mechanism.

The transcription factor NRF2 has been demonstrated to gate the antioxidant response (Baxter and Hardingham, 2016). It binds to the promoter element, the Antioxidant Response Element (ARE) located in antioxidant genes (Baxter and Hardingham, 2016). Under normal conditions, NRF2 is targeted by the endogenous inhibitor KEAP1 for degradation (Baxter and Hardingham, 2016). In the case of redox imbalance, KEAP1 undergoes oxidative modification, which prevents the degradation of NRF2 and promotes the transcription of the antioxidant genes, including those involved in the tripeptide glutathione (GSH) pathway (Baxter and Hardingham, 2016; Harvey et al., 2009).

As mentioned earlier, astrocytes' endfeet cover most of the brain capillaries’ surface (Schurhoff and Toborek, 2023). Positioned at this junction, astrocytes are among the first ones to encounter toxic compounds (Dringen et al., 2014). This makes astrocytes the first line of defence of the brain against harmful substances, capable of inducing oxidative stress (Dringen et al., 2014). In response, astrocytes export up to 10% of their GSH per hour (Baxter and Hardingham, 2016; Dringen et al., 2014).

Through this export, astrocytes contribute significantly to neuronal GSH levels, thereby supporting not only their own but also neurons’ antioxidant defence (Baxter and Hardingham, 2016). Various studies have shown higher GSH levels in astrocytes compared to neurons (Baxter and Hardingham, 2016). Astrocytes not only have higher NRF2 activity, but they also have a high capacity to produce and store GSH compared to neurons (Baxter and Hardingham, 2016). Co-culturing neurons with astrocytes shows double the amount of GSH available in neurons than when neurons are in the absence of astrocytes (Dringen et al., 1999; Pérez-Sala and Pajares, 2023). Moreover, cysteine, one of the amino acids limiting GSH availability in neurons, is provided by astrocytes (Dringen et al., 1999). Together, these findings highlight the importance of astrocytes in GSH availability.

To sustain the export of GSH and meet the ongoing demands of the antioxidant defence, continuous synthesis of GSH is required (Dringen et al., 2014). GSH is synthesised in astrocytic cytosol from glutamate, cysteine and glycine through two ATP-dependent enzymatic steps involving glutamate cysteine ligase and GSH synthetase (Fig. 3) (see Dringen et al., 2014 for more information) (Dringen et al., 2014; Pérez-Sala and Pajares, 2023). Once synthesised, GSH acts through multiple antioxidant mechanisms (Dringen et al., 2014). For example, it is a substrate of GSH-S-transferases (GSTs), which conjugate GSH to endogenous or exogenous electrophilic compounds for detoxification (Dringen et al., 2014).

### 7.1 Implications in Alzheimer's disease

Increasing GSH levels has been proposed as a potential therapeutic strategy to improve cognitive impairments associated with AD (Mandal et al., 2019). Diminished GSH synthesis could therefore be linked to the pathogenesis of AD (Haddad et al., 2021), as it potentially impairs detoxification processes. More generally, oxidative stress is associated with AD progression (Ionescu-Tucker and Cotman, 2021). High levels of ROS can promote the aggregation of Aβ42 into plaques and hyperphosphorylated tau into neurofibrillary tangles (the hallmarks of AD) (Ionescu-Tucker and Cotman, 2021).

Moreover, the ApoE genotype is shown to have an impact on oxidative stress and oxidative stress-mediated inflammation (Chico et al., 2013). Plasma from ApoE4 (AD risk factor) carriers seems to be more oxidised than non-ApoE4 carriers in AD (Fernandes et al., 1999).

### 7.2 Circadian rhythm and antioxidant defence

Interestingly, GSH-dependent detoxification in astrocytes appears to be influenced by circadian regulation. The basal GSH synthesis in astrocytes is regulated by NRF2 (He and Hewett, 2025). Additionally, the transcription of key enzymes in this synthesis, such as glutamate-cysteine ligase (GCL) and glutathione synthetase (GSH-syn) is regulated by NRF2/ARE (Pekovic-Vaughan et al., 2014). Notably, the NRF2/ARE pathway itself is regulated by the circadian clock (Pekovic-Vaughan et al., 2014). This suggests that GSH synthesis may be indirectly controlled by the circadian rhythm. In support of this, Pekovic-Vaughan et al. (2014) demonstrated that *Clock*^Δ19/Δ19^ mutated mice not only show lower GSH levels but also reduced NRF2 levels associated with increased protein oxidative damage.

Moreover, core clock protein BMAL1, which regulates the NRF2 pathway and its downstream targets (Bevinakoppamath et al., 2022; Sun et al., 2021), has been shown to bind to the E-box region of the NRF2/ARE promoter region, enhancing transcription of antioxidant genes (Bevinakoppamath et al., 2022; Sun et al., 2021). BMAL1-deletion leads to astrogliosis, accompanied by oxidative stress, shown to be at least in part mediated by the suppression of glutathione-S-transferase (GST) (Lananna et al., 2018). GSTs are essential in the detoxification of endogenous and exogenous compounds. A decline in GST levels leads to impairments in this detoxification response, causing oxidative stress, seen by increased ROS levels (Lananna et al., 2018).

GSH levels themselves show circadian oscillations, with an increase during the circadian night (Budkowska et al., 2022). All major oxidative stress enzymes were demonstrated to be under circadian regulation, except for GST activity (Budkowska et al., 2022). Although GST levels themselves do not show circadian oscillations, they are controlled by core clock components (i.e., BMAL1 and CLOCK) (Lananna et al., 2018); thus, indirect control cannot be ruled out and these effects may reflect direct transcriptional regulation instead of circadian control.

Given the role of oxidative stress in AD pathology (Ionescu-Tucker and Cotman, 2021), maintaining an effective antioxidant defence is crucial (Mandal et al., 2019). Incorrect functioning can lead to impaired detoxification of ROS, thereby exacerbating the AD phenotype (Ionescu-Tucker and Cotman, 2021). In line with this, disruption of core clock genes has been associated with reduced GSH, NRF2 (Pekovic-Vaughan et al., 2014) and GST (Lananna et al., 2018), increasing vulnerability to oxidative stress. Since neurons depend on astrocytic antioxidant support, its dysregulation appears to play a major role in AD pathology. Therefore, restoring redox balance via the astrocytic clock represents a promising future target of investigation.

## 8 Conclusion

Astrocytes are now recognised as integral mediators in the maintenance of central nervous system homeostasis. Specifically, astrocytes contribute to brain waste clearance and homeostasis through multiple mechanisms, including the blood-brain barrier, glymphatic system, glutamate levels, autophagy and oxidative defence. Many of these are individually controlled and affected by circadian clock gene mutations (Table 1). It remains unclear whether these genetic manipulations of clock genes lead to these effects via circadian disruptions or via other pleiotropic routes. Moreover, most of these studies are done in mice and one should be cautious in translating these directly to the human system. Astrocytic clock gene alterations, including *Bmal1* knockout, *Clock* mutations, *Per*- and/or *Cry*-null models, are associated with changes in several brain waste clearance and homeostatic pathways. Among these, the blood-brain barrier, glutamate regulation and the antioxidant defence impact Alzheimer's pathology compromising neuroprotection and brain waste clearance upon clock disruption. In contrast, some clock-gene disruptions are associated with increased autophagy-related markers and *Aqp4* expression, which may represent compensatory responses; however, whether these changes improve functional clearance or influence AD pathology remains uncertain. A key unresolved question is whether astrocytic clock disruption alone is sufficient to substantially alter brain waste clearance *in vivo*. Astrocytic clock's direct contributions to brain waste clearance mechanisms might be redundant due to compensatory mechanisms through interactions with other cell types or the influence of other components. Sleep/wake state represents an important potential confound when interpreting circadian effects on brain waste clearance. Sleep is a major regulator of brain waste clearance and is itself influenced by astrocytic function (Jackson et al., 2019). Because sleep and circadian timing are physiologically intertwined, much of the evidence discussed in this review cannot fully exclude a contribution from sleep/wake state. Rather than treating sleep as outside the scope of this review, we emphasize that disentangling circadian clock-driven and sleep-driven contributions to brain waste clearance is a critical open question for the field.

**Table 1:** Qualitative summary of evidence for astrocyte-clock-regulated homeostatic and clearance pathways relevant to Alzheimer's disease This table summarizes the strength of evidence linking astrocyte-associated clearance and homeostatic pathways to circadian or (astrocytic) clock-gene-dependent regulation, their relevance to AD models or pathology, the proposed direction of effect after clock disruption, and remaining knowledge gaps.

Pathway/Route	Role Astrocytes	AD relevance	Circadian rhythmicity	Clock-gene dependence	Astrocytic-clock	Disruption of the clock	AD model	Key knowledge gaps
**Neurotransmitter homeostasis**	Part of tripartite synapse; GLAST/GLT-1 mediated glutamate uptake (Costa and Montagnese, 2021)	↑ Extracellular glutamate → neuronal hyperexcitation, neurotoxicity & AD-associated cognitive impairment (Keimasi et al., 2023; Mahmoud et al., 2019; Wang and Reddy, 2016), ↓ GLAST/GLT-1 → neurotoxicity & neurodegenerative characteristics (Rothstein et al., 1996; Takahashi et al., 2015)	**+** Astrocytes, extracellular glutamate levels, glutamine synthetase and CX43 show rhythmic oscillations (Brancaccio et al., 2019; Costa and Montagnese, 2021; Jackson et al., 2019; Leone et al., 2015)	**?**	**+** Astrocyte-specific *Cry1* expression in Cry1/2-null mice → rescue of circadian behavior and extracellular glutamate rhythms (Brancaccio et al., 2019), Pan-astrocytic *Bmal1* deletion → **↓** GABA transporters (Barca-Mayo et al., 2017; Hastings et al., 2023)		**?**	Cell-autonomous vs. exogenous control of GLAST/GLT-1 rhythms, whether clock-driven CX43 and/or glutamine synthetase alterations alter extracellular glutamate oscillations *in vivo*
**BBB**	Part of NVU endfeet; ion and transporter regulation (Cabezas et al., 2014; McConnell and Mishra, 2022; Schurhoff and Toborek, 2023)	↑ BBB breakdown in AD. ApoE4 astrocytes → ↓ BBB integrity & ↑ Aβ42 accumulation, ↑ P-gp → ↑ BBB's selectivity (R. J. Jackson et al., 2021; Nishitsuji et al., 2011)	**++** Tight junction proteins, ApoE, AQP4, P-gp show circadian rhythmicity (Hablitz et al., 2020; Kervezee et al., 2014; McKee et al., 2022)	**–** Absence of BMAL1 → **↓** cortical AQP4 (Nakazato et al., 2017)	**+** *Bmal1* aKO → ↑ *Apoe* and *Aqp4* mRNA (Lananna et al., 2018; McKee et al., 2019)		**?**	Discrepancy of *Aqp4* mRNA vs protein, clock-driven P-gp *in vivo*, astrocyte-specific clock vs non-circadian BMAL1 effects
**Glymphatic System**	AQP-4 mediated CSF-ISF exchange and waste clearance (Iliff et al., 2012)	Glymphatic dysfunction/AQP4 impairment → ↑ Aβ42 (Silva et al., 2021)	**+** Circadian control of glymphatic influx (including AQP4), solute clearance and CSF-drainage (Hablitz et al., 2020)	**?**	**–** *Bmal1* aKO → ↑ *Aqp4* mRNA expression (Lananna et al., 2018)		**?**	Effect of astrocyte-intrinsic clocks, independent of sleep/wake state and BP, on glymphatic clearance *in vivo*, AQP4's influence on AD phenotype
**Autophagy***(CMA & Macroautophagy)*	Aβ42 and tau uptake & lysosomal degradation (Gomez-Arboledas et al., 2017; Ries and Sastre, 2016; Wyss-Coray et al., 2003)	Autophagy impairment → ↑ Aβ42/tau & neurotoxicity (Barmaki et al., 2023)	**+** Soluble Aβ peptides show circadian rhythmic activity (Kang et al., 2009; Kress et al., 2018); intrinsic vs systemic control unclear	**?**	**+** Astrocyte-specific *Bmal1* deficiency → ↑ endocytosis, ↑ LAMP1, ↑RAB7 (McKee et al., 2023), ↑ BAG3 and autophagosomes (Sheehan et al., 2023), ↑ mTOR activity (Khapre et al., 2014)		Astrocyte *Bmal1* deletion → **↓**tau pathology via *Bag3* without changing Aβ42 plaque burden (Sheehan et al., 2023)	Autophagy contribution to Aβ42 rhythmic fluctuations*,* basal vs rhythmic CMA regulation by BMAL1. Functional consequences of ↑ mTOR activity for Aβ42 accumulation, causality between clock regulation and autophagic activity
**Oxidative Stress & Antioxidant Defense**	GSH-export; astrocytic and neuronal antioxidant support (Baxter and Hardingham, 2016)	ROS → Aβ/tau pathology (Ionescu-Tucker and Cotman, 2021); ↑ GSH potentially protective for AD (Mandal et al., 2019)	**++** GSH & NRF2/ARE show circadian oscillations (Budkowska et al., 2022; Pekovic-Vaughan et al., 2014)	**+** Clock^Δ19/Δ19^ mutated mice → **↓** GSH and NRF2 (Pekovic-Vaughan et al., 2014) BMAL1 binds directly to the NRF2/ARE promoter region (Bevinakoppamath et al., 2022)	**?**		*Bmal1* aKO ✕ APP/PS1 mice → altered **neuroinflammation**, no change in Aβ42 plaque burden (McKee et al., 2022)	Astrocyte-clock control of GSH/GST levels, direct vs indirect/non-rhythmic clock regulation of GST, consequences for AD pathology

*Explanation of symbols*.

• **(++) Established effect:** Robust circadian regulation (∼24h oscillations) and/or direct evidence that astrocyte-clock manipulation causes a functional change.

• **(+) Moderate effect:** Circadian fluctuations and/or effects of core clock gene disruption are reported, but results are inconsistent, confounded by systemic/exogenous factors or may reflect non-circadian effects of clock genes.

• **(−) No/limited effect:** Circadian fluctuations are minor or inconsistent, and clock disruption produces no functional effect or cell-autonomous regulation has been ruled out.

• **(?) Not investigated/speculative:** Circadian or astrocyte-clock regulation is hypothesized but direct empirical evidence is missing.

• () Protective effect of the pathway/route upon clock disruption.

• () Harmful effect of the pathway/route upon clock disruption.

• () The pathway/route can have both a protective and harmful effect upon clock disruption.

***Abbreviations***: AD, Alzheimer's disease; Aβ, amyloid-beta; aKO, astrocyte-specific knockout; Apoe: apolipoprotein E; APP/PS1, amyloid precursor protein/presenilin-1; AQP4, aquaporin-4; BAG3, BCL2-associated athanogene 3; BBB, blood-brain barrier; Bmal1, Brain and Muscle Arnt-like protein 1; Clock, circadian locomotor output cycles kaput; CMA, chaperone-mediated autophagy; Cry, cryptochrome; CSF, cerebrospinal fluid; Cx43, connexin43; GLAST, glutamate-aspartate transporter; GLT-1, glutamate transporter-1; GSH, glutathione; GST, glutathione-S-transferase; ISF, interstitial fluid; KO, knockout; LAMP1, lysosomal-associated membrane protein 1; mTOR, mammalian target of rapamycin; NVU, neurovascular unit; P-gp, permeability glycoprotein; RAB7, Ras-related protein Rab-7a; ROS, reactive oxygen species.

Adding another layer of complexity is the notion that AD is associated with an imbalance in Aβ42/Aβ40, aggregation of Aβ42, tau tangle formation and several risk factors such as the *ApoE4* gene. Most studies focus on Aβ42 aggregation and therefore it is unclear whether tau is affected or not. ApoE4 negatively affects multiple brain clearance and homeostatic pathways, from its core role in lipid and cholesterol transport to downstream effects on the blood-brain barrier (Zhou et al., 2023), autophagy (Simonovitch et al., 2016), and oxidative stress (Chico et al., 2013; Fernandes et al., 1999). Although it is a genetic risk factor, interestingly enough, the expression of *ApoE4* is under the control of the astrocytic circadian clock (Lee et al., 2023). Moreover, the ApoE4 isoform in mice alters circadian rhythm activity, resulting in a different behavioural phenotype of activity compared to the wild-type mice (Graybeal et al., 2014).

Although previous research has examined the relationship between the circadian clock, astrocytes, brain waste clearance and AD, few studies have directly integrated these domains. Specifically, the direct connection between the astrocytic clock to brain waste clearance mechanisms and their implications for Alzheimer's disease pathology remains poorly understood. This review suggests that there is a link between these domains. The discovery of astrocytes' specific importance in encoding circadian information offers a new perspective on AD development and progression. However, ambiguities remain regarding the extent to which the astrocytic clock influences overall brain waste clearance. Currently, it is unclear whether the effects observed in astrocytic clock disruption models reflect true circadian control or normal transcriptional control. Moreover, it is difficult to distinguish cause from consequence. Additionally, the extent to which imbalance of these mechanisms contributes to Alzheimer's pathology remains unclear. Important questions and shortcomings that deserve attention are: inducible, astrocyte-specific clock manipulations in adult AD models to avoid developmental confounds; time-of-day-resolved sampling of Aβ and tau clearance; direct *in vivo* measurement of glymphatic flow rather than reliance on AQP4 expression as a proxy; experimental designs that explicitly control for or dissociate sleep/wake state from circadian phase; comparisons across *ApoE* genotypes; and human studies incorporating circadian phase alongside established AD biomarkers. Future research should aim to resolve these uncertainties and further investigate the intersection of the astrocytic circadian clock, brain waste clearance and Alzheimer's disease. This could reveal important insights into early-stage mechanisms of AD as well as novel therapeutic targets. These connections help us gain an understanding of the molecular mechanisms and their interactions underlying AD, which could enable early detection of pathological changes, potentially improving treatment efficacy and patient outcomes.

## CRediT authorship contribution statement

**Chun Lei Koenders:** Writing – original draft, Investigation, Conceptualization. **Roland Chiu:** Writing – review & editing. **Tiago Medeiros-Furquim:** Writing – review & editing. **Steven Bergink:** Writing – review & editing, Supervision.

## Declaration of competing interest

The authors declare that they have no known competing financial interests or personal relationships that could have appeared to influence the work reported in this paper.

## Data Availability

No data was used for the research described in the article.
